# ChemGAPP: a tool for chemical genomics analysis and phenotypic profiling

**DOI:** 10.1093/bioinformatics/btad171

**Published:** 2023-04-04

**Authors:** Hannah M Doherty, George Kritikos, Marco Galardini, Manuel Banzhaf, Danesh Moradigaravand

**Affiliations:** Institute of Microbiology and Infection and School of Biosciences, University of Birmingham, Birmingham B15 2TT, United Kingdom; European Molecular Biology Laboratory, Genome Biology Unit, Heidelberg 69117, Germany; Institute for Molecular Bacteriology, TWINCORE Centre for Experimental and Clinical Infection Research, A Joint Venture Between the Hannover Medical School (MHH) and the Helmholtz Centre for Infection Research (HZI), Hannover 30625, Germany; Cluster of Excellence RESIST (EXC 2155), Hannover Medical School (MHH), Hannover 30625, Germany; Institute of Microbiology and Infection and School of Biosciences, University of Birmingham, Birmingham B15 2TT, United Kingdom; KAUST Smart-Health Initiative and Biological and Environmental Science and Engineering (BESE) Division, King Abdullah University of Science and Technology (KAUST), Thuwal, Makkah 23955-6900, Saudi Arabia; KAUST Computational Bioscience Research Center (CBRC), King Abdullah University of Science and Technology (KAUST), Thuwal, Makkah 23955-6900, Saudi Arabia

## Abstract

**Motivation:**

High-throughput chemical genomic screens produce informative datasets, providing valuable insights into unknown gene function on a genome-wide level. However, there is currently no comprehensive analytic package publicly available. We developed ChemGAPP to bridge this gap. ChemGAPP integrates various steps in a streamlined and user-friendly format, including rigorous quality control measures to curate screening data.

**Results:**

ChemGAPP provides three sub-packages for different chemical-genomic screens: ChemGAPP Big for large-scale screens; ChemGAPP Small for small-scale screens; and ChemGAPP GI for genetic interaction screens. ChemGAPP Big, tested against the *Escherichiacoli* KEIO collection, revealed reliable fitness scores which displayed biologically relevant phenotypes. ChemGAPP Small demonstrated significant changes in phenotype in a small-scale screen. ChemGAPP GI was benchmarked against three sets of genes with known epistasis types and successfully reproduced each interaction type.

**Availability and implementation:**

ChemGAPP is available at https://github.com/HannahMDoherty/ChemGAPP, as a standalone Python package as well as Streamlit applications.

## 1 Introduction

The field of chemical genomics and high-throughput phenomic profiling has revolutionized the ability to functionally annotate unknown genes on a genome-wide level. With applications for drug discovery, mechanism of action studies, and conditional essentiality studies, chemical genomics is a rapidly growing field (Brochado and Typas 2013; Klobucar and Brown 2018). Chemical genomic screens systematically assess the effect of chemical or environmental perturbations (stresses) on single-gene mutant libraries. The resulting phenotypes can range from colony size as a proxy of fitness, colour uptake to quantify biofilm formation, and changes in colony topology to assess biofilm morphology (Kritikos et al. 2017). Individual observations can be studied in isolation, based on the observed phenotype of a gene in a specific condition, thereby creating a functional link between a stress and a defined genetic perturbation. However, the true power of chemical genomic approaches lies within the ability to calculate phenotypic profiles for each mutant based on their phenotypes across conditions (Nichols et al. 2011). These phenotypic profiles can be hierarchically clustered to reveal similarities between mutant profiles to functionally cluster genes or stress conditions. This linkage reconstitutes biological pathways and complexes and therefore can functionally cluster unknown genes to known biology (Nichols et al. 2011). The power of chemical genomic screens, and the plethora of valuable information they produce, has led to many important biological discoveries and their popularity in the field of systems microbiology (Tong et al. 2001; Schuldiner et al. 2005; Gomez and Neyfakh 2006; Fajardo et al. 2008; Tamae et al. 2008; Typas et al. 2010; Nichols et al. 2011; Koo et al. 2017; Bobonis et al. 2022).

Despite the rise in chemical genomics studies, there are currently no dedicated programs to analyse the type of data these screens produce. Currently, a variety of methods are being implemented for the analysis of data and are being performed by in-house scripts or are adaptations of packages used for similar techniques (Collins et al. 2006; Dixon et al. 2009; Nichols et al. 2011; French et al. 2016; Shiver et al. 2016, 2021). A number of image analysis software are available for converting phenotypes within plate images into numerical values, such as gitter or Iris; however, these are unable to convert these values into fitness scores (Wagih and Parts 2014; Kritikos et al. 2017). Another software that has been frequently implemented, which is able to produce fitness scores, is EMAP Toolbox (https://sourceforge.net/projects/emap-toolbox/); however, this tool is now deprecated (Collins et al. 2006; Collins, 2006; Nichols et al. 2011). Furthermore, EMAP was originally developed to handle genetic interactions within yeast and henceforth does not offer a standard solution for handling data from assays under conditions. None of these options are accessible for users with limited computational expertise, as many require the knowledge of coding languages such as R or MATLAB to implement (French et al. 2016; Shiver et al. 2016, 2021). Consequently, there is a clear gap that hinders researchers from effectively using chemical genomics approaches. This study, therefore, introduces an easy to use, new wrapper software and Streamlit app called ChemGAPP (*Chem*ical *G*enomics *A*nalysis and *P*henotypic *P*rofiling), which has been developed as a comprehensive chemical genomics data analysis software.

Here, we provide evidence for ChemGAPP’s effectiveness to reliably analyse chemical genomics data. By providing ChemGAPP it will enable the wider scientific community to use chemical genomics approaches to reveal significant, biologically relevant insights into gene function, drug mechanisms of action, and antibiotic resistance mechanisms.

## 2 Materials and methods

**Figure 1:**
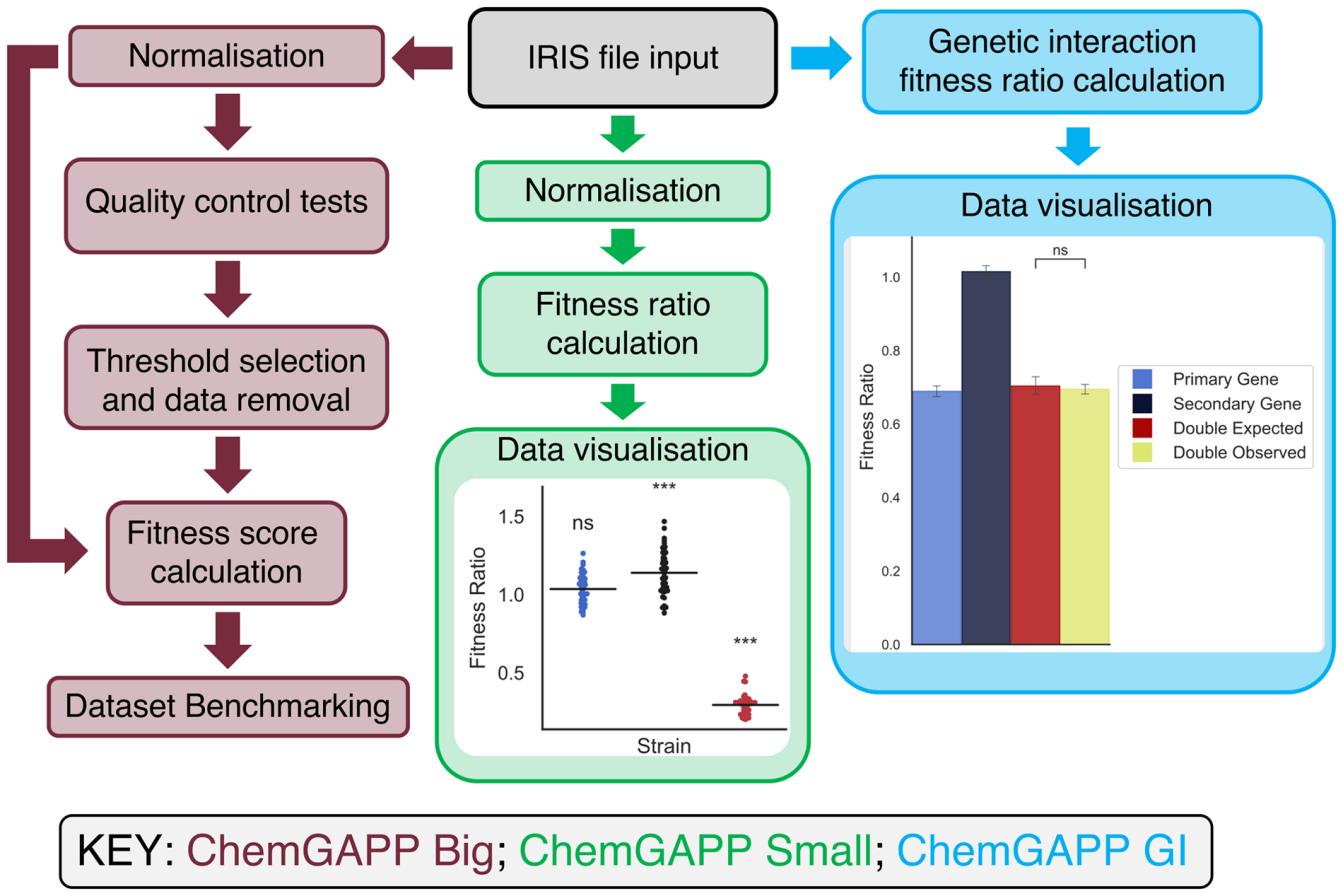
Workflow of the ChemGAPP packages. ChemGAPP Big analyses large scale chemical genomic screens. ChemGAPP Small analyses small scale chemical genomic screens. ChemGAPP GI analyses small scale genetic interaction screens.

ChemGAPP comprises of three pipelines ChemGAPP Big, ChemGAPP Small, and ChemGAPP GI, each specifically designed for different types of chemical genomic screens (Fig. 1).

### 2.1 ChemGAPP Big pipeline

In order to validate the ChemGAPP Big pipeline, we used the chemical genomic screen data from Nichols et al. (2011) that was reanalysed using Iris by Kritikos et al. (2017) and Nichols et al. (2011). Here, Kritikos et al. (2017) measured colony integral opacity as the colony size measure, since this represents 3D colony growth. The Nichols et al. study was the first major chemical genomic screen within bacteria and was performed in *Escherichia coli*. Nichols et al. assessed the phenotypes of the KEIO collection, an in-frame single-gene knockout mutant library in *E.coli* K-12, within over 300 conditions (Baba et al. 2006; Nichols et al. 2011). The Nichols et al. study successfully produced >10 000 phenotypes, categorized various biologically relevant hits and suggested functions for genes with previously unknown function (Nichols et al. 2011).

#### 2.1.1 ChemGAPP Big: conversion of Iris files

ChemGAPP Big processes all classical stages of data analysis within large scale chemical genomic screens, whilst introducing new features to improve upon this analysis. The first stage is to produce a dataset from the experimental data. Initial quantification of screening plates is increasingly performed by the image analysis software Iris (Kritikos et al. 2017). Iris’s popularity is due to its versatility in phenotype measurements, analysing many phenotypes, including size, integral opacity, circularity, colour, etc. (Kritikos et al. 2017). Therefore, the Iris file format was chosen as the default input for ChemGAPP. Despite this, there is no limit to the usage of other image analysis software, as long as the data is compiled into the Iris file format. The Iris files were compiled into a dataset comprising of each condition replicate plate as the columns and colony size data as the values. To initially improve the dataset, zero values where not all replicates were also zero were removed, since these likely represent mis-pinned colonies.

#### 2.1.2 ChemGAPP Big: plate normalization

ChemGAPP Big then removes noise from the experimental data and scales data to become comparable via a two-step normalization (Supplementary Fig. S1). It is vital to perform plate normalization before the data can be scored due to the noise that arises during large chemical screens. A major issue within chemical genomic screens is the edge effect, which is targeted by the first stage of normalization (Baryshnikova et al. 2010). Since mutants are densely pinned on plates, the outermost colonies are left with more space. This results in increased growth around the plate edges, due to reduced nutrient competition (Baryshnikova et al. 2010). The aim of the first step of normalization is therefore to reduce this intrinsic noise. The second stage standardizes all plates such that regardless of condition, the median colony sizes are comparable. This is important for the generation of mutant S-scores across different conditions later in the pipeline.

Plate normalization was performed as in Collins et al. (2006), with the addition of an initial check for edge effects to evaluate if the first normalization step was required. A Wilcoxon rank sum test was performed between the colony size distributions for the outer two edges and the centre of the plate. If edge effects are present, the distributions will differ, and the first stage of normalization will be performed. In the second stage, all plates were scaled such that the plate middle mean was equal to the median colony size of all mutants within the dataset.

#### 2.1.3 ChemGAPP Big: quality control analyses

Within ChemGAPP Big, we have implemented multiple quality control tests to find common errors which occur within chemical genomic screens. Acquiring the physical raw data for these screens is challenging as often thousands of plates need to be accurately processed. The most common errors which occur in the laboratory are mislabelled plates, inverted images, and mutants being unequally pinned between replicates or missed entirely. In addition, despite Iris’s ability to robustly quantify most mutants, its detection algorithm can fail and create artefacts. The user can choose which tests to implement, allowing users to employ the right tests for the issues that have arisen within their datasets.

##### 2.1.3.1 Quality control analyses: Z-score test

The first test, aimed at distinguishing between the phenotype of no growth and artefacts due to mis-pinned colonies or missed detection by Iris, is the Z-score analysis. The Z-score value describes the distance of a test value from the population mean in measures of standard deviation (std). The distribution of the population is adjusted to a standard normal distribution, with a mean of zero and a std of 1 (Driscoll et al. 2000). The Z-score test compares replicate colonies for each plate in each condition and highlights outlier colonies (Z-score value >1 or <−1) or missing values. ChemGAPP Big then calculates percentage normality for each replicate plate, i.e. the percentage of colonies within the plate which are not outliers or missing values.

##### 2.1.3.2 Quality control analyses: Mann–Whitney test

The Mann–Whitney test compares the distribution of colony sizes between replicate plates. If two replicate plates are reproducible, they should fall into the same distribution. This test is particularly useful for identification of mislabelled plates since distributions of colonies will significantly differ. Furthermore, the Mann–Whitney test can highlight unequal pinning effects. For each replicate plate in the condition of interest, the distribution of mutant colony sizes was compared to each other replicate’s distribution and a Mann–Whitney *P*-value calculated. The mean *P*-value for each replicate was calculated by averaging the Mann–Whitney *P*-values for comparisons including the replicate of interest.

##### 2.1.3.3 Quality control analyses: condition level tests

Where all replicate plates are non-reproducible, a condition can be deemed unsuitable for use. This can occur, for example, when a condition produces a coloured media which is difficult to distinguish from the colonies. Here, Iris can struggle to differentiate the colonies from the background media, leading to artefacts in detection (Kritikos et al. 2017). Therefore, it is important to consider if whole conditions should be removed from the dataset. Here, two tests were implemented, the first calculated the variance in the Mann–Whitney *P*-values for each replicate plate. A large variance here suggests that the entire condition may be detrimental, since it is likely all replicate distributions differ from each other. Finally, a general variance analysis was employed. Here, the variance of each replicate colony in the condition of interest was calculated. The mean of these variances was then calculated to gain an average variance across all plates for each condition. A high variance implies that most colony replicates differ within their size, and thus the entire condition is non-reproducible.

#### 2.1.4 ChemGAPP Big: threshold selection and data removal

Curation of the dataset is a fine balance between removing detrimental data and remaining informative. Therefore, the thresholds at which data are removed are left to the user. However, ChemGAPP Big allows for an informed choice by outputting a visual representation of the quantity of data lost at different thresholds for the various tests (Supplementary Fig. S2). For the Z-score and Mann–Whitney tests, the percentage of plates lost within each condition at certain thresholds was calculated. Whereas, for the condition level analyses, the overall number of conditions that would be lost at different thresholds was calculated. For Z-score analysis data, thresholds of 20%, 30%, 40%, 50%, 60%, 70%, and 80% for percentage normality were tested. For the Mann–Whitney test and the condition level tests, thresholds were generated based on the obtained range of test values. The chosen thresholds for the curated dataset were 80%, 0.07585, and 0.0128, for normality, Mann–Whitney plate analysis, and Mann–Whitney condition analysis. Data were not removed based on the average variance test since little variance within conditions was observed (Supplementary Fig. S2). Chosen thresholds were inputted and a new dataset with the failing plates and conditions removed was outputted and normalized.

#### 2.1.5 ChemGAPP Big: fitness score calculation

In order to find meaning within the data, fitness scores must be produced. Within ChemGAPP Big, this is performed using the S-score test. The S-score test, as described in Collins et al. (2006), is a modified *T*-test where the mean of a set of colony replicates, for the desired mutant within a specific condition, are compared to the median size of that mutant across all conditions. This provides a statistical score for fitness, with positive scores showing increased fitness and negative scores showing decreased fitness. Comparing a mutant to itself across all conditions as a simulated wildtype is more powerful than comparing to the true wildtype. This is because a mutant may have generally increased or decreased fitness than the wildtype. By comparing to itself, a true baseline for comparison is used. Furthermore, since S-scores correlate to statistical significance, they provide a robust and informative measurement of how strong a phenotype is across various conditions.

Where S-scores were calculated as infinity, scores were converted to missing values. The S-scores of each plate were then scaled such that the interquartile range (IQR) of the scores was equal to 1.35. This scales the datasets such that the values for significant hits are for S-scores <−3 and >2. In general, the majority of S-scores within a dataset will be close to zero. Therefore, significant hits are S-scores that are classified as outliers. Outliers are commonly determined as scores deviating from the first or third quartiles by 1.5 times the IQR, which in this case is ∼2. Therefore, significant scores would be classified as <−2 and >2. However, since loss of function mutants are more common, a higher threshold of deviation was chosen to represent significant negative S-scores. The final non-curated dataset was visualized within Treeview3 (Keil et al. 2018). Data were hierarchically clustered for rows and columns using uncentred Pearson Correlation and average linkage.

#### 2.1.6 ChemGAPP Big: benchmarking the curated dataset

Following dataset curation, it is vital to confirm which dataset has the most accurate fitness scores. In order to measure the accuracy of the datasets, the concept that genes within the same operon often have a similar function was drawn upon. If genes are functionally related, we would expect their knockout mutants to behave similarly across the different tested conditions. Thus, they would have similar phenotypic profiles, which is their set of S-scores across all conditions (Nichols et al. 2011). Therefore, the non-curated and curated datasets were compared to each other as well as to the Nichols et al. (2011) KEIO dataset, which was analysed within EMAP Toolbox. Genes from the same operon and genes from differing operons were subjected to cosine similarity analysis. Phenotypic profiles that are similar will have cosine similarity scores close to 1, those with no similarity will have scores close to zero and negatively correlated profiles will have scores close to −1. Confusion matrices were produced at different thresholds of cosine similarity scores, ranging from −1 to 1, by increments of 0.1. Here, a true positive was a set of genes from the same operon with a cosine similarity score greater than the threshold; a true negative was a set of genes from different operons with a cosine similarity score lower than the threshold; a false positive was a set of genes from different operons with a cosine similarity score greater than the threshold; and a false negative was a set of genes from the same operon with a cosine similarity score lower than the threshold. At each threshold, the sensitivity, specificity, and false positive rate were calculated and plotted in a receiver operating characteristic curve. The area under the curve (AUC) for the datasets was then calculated and compared.

#### 2.1.7 ChemGAPP Big: production of bootstrapped dataset

In order to evaluate the accuracy and robustness of the S-scores produced by ChemGAPP Big, a bootstrapped dataset was produced. The KEIO original and curated normalized datasets were bootstrapped 1000 times with replacement. Each of the 1000 datasets was subjected to S-score analysis. The mean of the bootstrapped S-scores for each mutant in each condition, were compared to the true S-scores and the mean absolute error (MAE) calculated.

### 2.2 ChemGAPP Small pipeline

In large scale chemical genomics, by design, the screens are unbiased, and the aim is to test mutant libraries against a manifold of stresses. Since in most conditions mutants are unlikely to display a phenotype, a normal distribution of colony sizes is achieved. In contrast in small scale studies a few or only one condition are screened, and, in such analyses, a confined hypothesis is often being tested. When testing a specific hypothesis, conditions are often specifically chosen with the assumption that there will be an associated phenotype. Therefore, the conjecture about the normal distribution of fitness effects across conditions may not hold. In these instances, ChemGAPP Big is not fit for purpose.

Therefore, we developed ChemGAPP Small for the analysis of targeted small-scale chemical genomics studies. In ChemGAPP Big fitness values are computed based on a minimum of ∼10 conditions with normally distributed colony sizes. In contrast, ChemGAPP Small generates fitness ratios for mutants versus the wildtype on the corresponding condition, in place of S-scores, without relying on any assumption about fitness distributions across conditions. By doing so, ChemGAPP Small allows users to robustly analyse smaller datasets without any constraints on gene or condition number. The small-scale Iris files were compiled and normalized as for ChemGAPP Big; however, zero values were not converted to missing values. Fitness ratios were computed for each plate, either by dividing the mean mutant colony size by the mean wildtype colony size for the bar plots and heatmap, or by dividing each individual colony by the mean wildtype colony size for the swarm plots. Significance for bar plots was measured by 95% confidence interval, whereas a one-way Analysis of Variance (ANOVA) was used to determine significant differences from the wildtype within the swarm plots.

### 2.3 ChemGAPP GI pipeline

The final package ChemGAPP GI (ChemGAPP *G*enetic *I*nteractions) can analyse genetic (epistatic) interaction screening data. Genetic interaction studies aim to determine the type of epistasis between two genes. ChemGAPP GI analyses single or multiple gene pairs on the same condition plate, producing separate plots for each gene pair. Genetic interactions are defined as instances where the observed fitness of a double knockout mutant is significantly different to the expected fitness ratio (Mani et al. 2008). This can be further categorized into two types, positive (alleviating), and negative (synergistic). In positive epistasis, the double knockout is fitter than anticipated and in negative epistasis, it shows decreased fitness (Mani et al. 2008).

ChemGAPP GI assesses the fitness of two single knockout mutants and a double knockout mutant, versus a wildtype, to calculate the observed and expected fitness ratios. By doing so, the tool provides a proxy for the strength of the genetic interactions and the mode of epistasis between the two genes. Genetic interaction Iris files were inputted into ChemGAPP GI and genetic interaction fitness ratios calculated. For the single and double knockouts, fitness ratios were calculated by dividing the colony size of the mutant by the colony size of the WT for that replicate. The double knockout expected fitness ratio was calculated by the formula:


Double expected=(ΔA colony size)/(WT colony size)*(ΔB colony size)/(WT colony size)

Significance was measured by 95% confidence interval error bars and one-way ANOVAs between the double observed and double expected fitness ratios.

## 3 Results

### 3.1 ChemGAPP Big identifies common errors within chemical genomic screens

**Figure 2:**
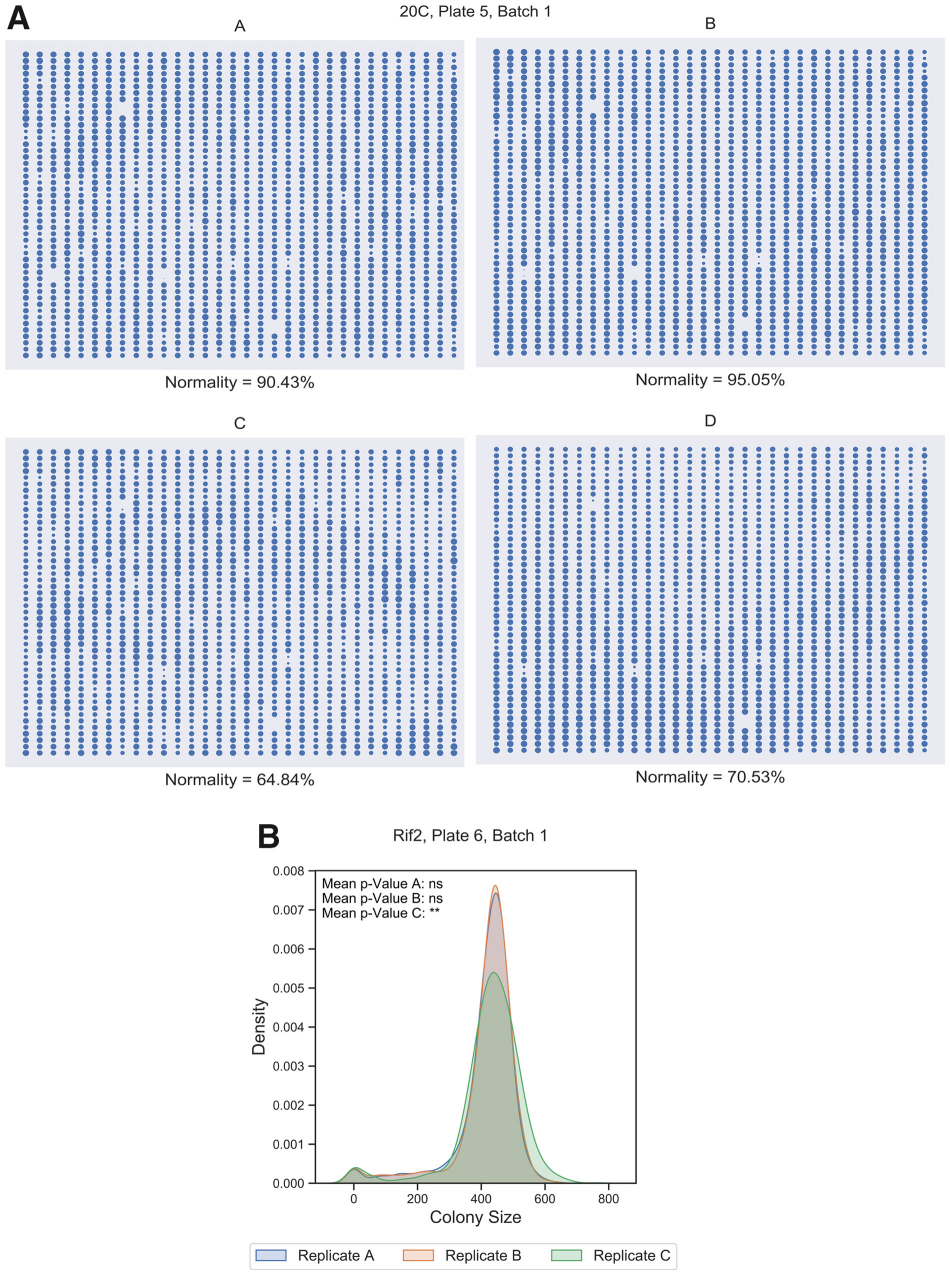
ChemGAPP Big highlights plates with errors common to chemical genomic screens. (A) Plate matrix depicting the colony sizes within replicate plates of the condition 20C (cold shock 20°C) and the percentage normality determined by the Z-score test. (B) Density of colony sizes for replicates A, B, and C for condition Rif2 (Rifampicin 2 µg/ml), Plate 6, Batch1. Difference in the distribution of C versus A and B is statistically significant by Mann–Whitney test. **0.001 < *P*-value ≤0.01; ns, non-significant.

In order to tailor ChemGAPP Big to large-scale chemical genomic screen data, we designed the quality control steps to target common errors that arise within the field. One frequent error in chemical genomic screens is the introduction of plate effects due to unequal pinning. To identify this, we conducted the Z-score test and looked at plates with low percentage normality. Two replicate plates C and D in the 20°C cold shock condition, had a reduced normality percentage of 64.84% and 70.53%, respectively (Fig. 2A). Figure 2A represents the colony sizes of the replicate plates. Conversely to C and D, Plates A and B had high percentage normality scores of 90.43% and 95.05%, respectively (Fig. 2A). Unequal pinning can be observed within Plates C and D. Within D, the colonies are consistently smaller in the upper segment of the plate and consistently larger in the lower segment. However, in C, it is the outer segments of the plate, excluding the corners, with reduced colony sizes, and the inner segment with increased sizes. Within Plates C and D, 17.9% and 15.69% of colonies were classified as larger than the mean replicate colony size, and 17.19% and 13.74% of colonies were smaller, respectively, reflecting what is seen in Fig. 2A.

To further detect unequal pinning, as well as missed pinning or undetected colonies, the Mann–Whitney test was performed. These effects are evident in Fig. 2B, where we identified a condition, i.e. 2 µg/ml rifampicin, in which one replicate (C) differed in its distribution of colony sizes. Replicate C had a mean Mann–Whitney *P*-value of 0.0018 (Fig. 2B). The distribution of C shown in Fig. 2B shows an increased number of larger and smaller colonies than A or B. Furthermore, C had more missing colonies (21) than A or B, which both had only 4 missing values (Supplementary Fig. S3). This is likely due to colonies being missed by pinning or by Iris detection, showing the ability of the tool to successfully pinpoint non-reproducible replicate plates, based on a variety of defects.

**Figure 3:**
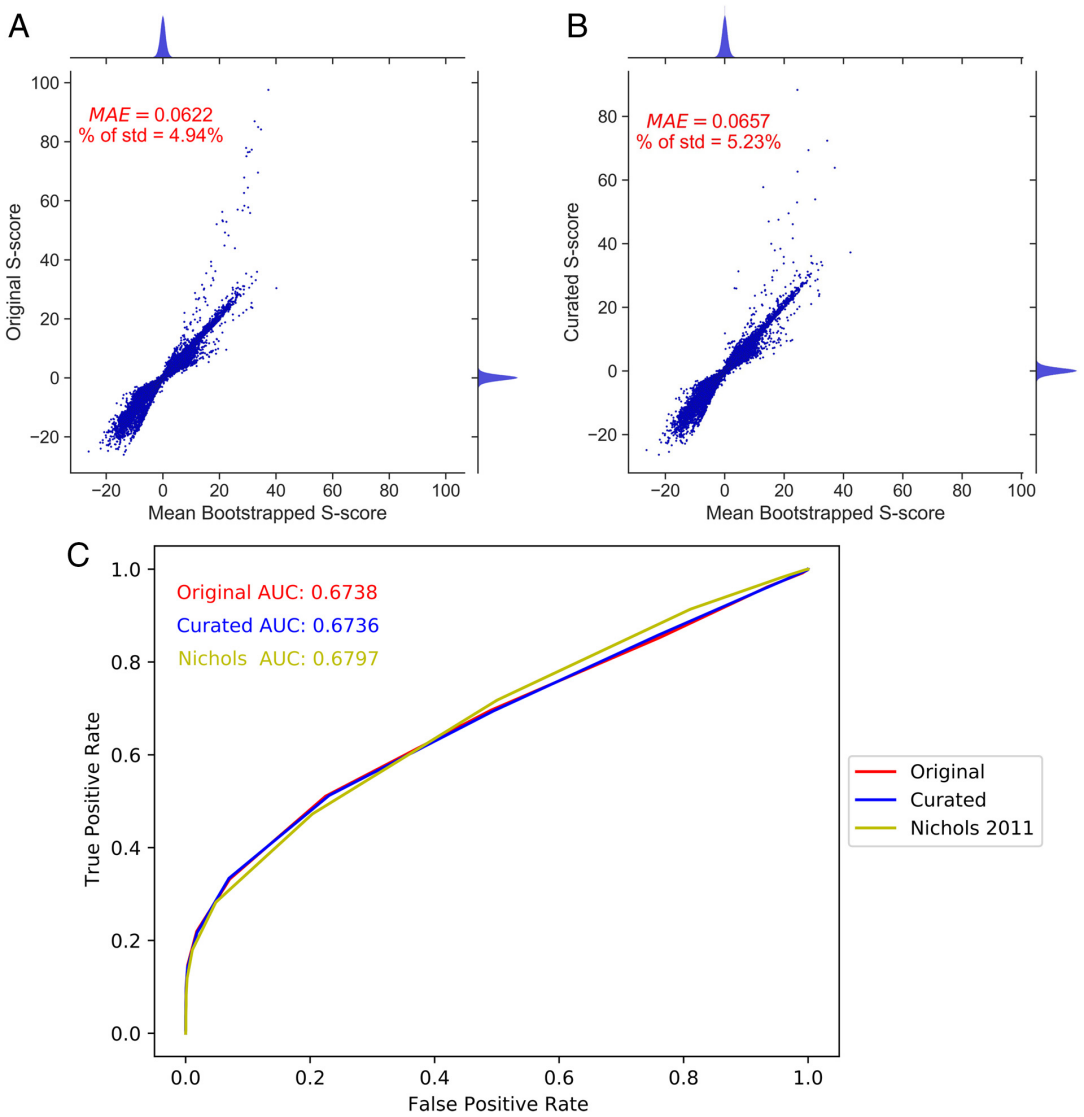
ChemGAPP produces robust and accurate S-scores. (A and B) Joint scatter and density plot depicting the difference between (A) the original non-curated S-scores versus the mean bootstrapped S-scores for each mutant within each condition of the KEIO dataset. (B) The curated S-scores versus the mean bootstrapped S-scores for each mutant within each condition of the KEIO curated dataset. Density plots show distribution of hits with various S-scores. For ease of visualization, outlier bootstrapped S-scores >125 were excluded, representing a negligible 0.000018% (A) and 0.000026% (B) of all values (see Supplementary Additional File S2). Outlier curated S-scores >125 were also excluded, representing a negligible percentage of all values (0.00056%). MAE, mean absolute error; % of std, percentage of the standard deviation for the original dataset that the MAE constitutes. (C) Receiver operating characteristic curve with AUC values for the KEIO ChemGAPP Big non-curated dataset (red line), the KEIO ChemGAPP Big curated dataset (blue line), and the KEIO dataset from Nichols et al. (2011) (yellow line).

### 3.2 S-scores are reliable and accurate representations of fitness

In order to evaluate if the S-scores produced by ChemGAPP Big were accurate and robust representations of fitness, a bootstrapped dataset was produced. The MAE for the non-curated dataset was 0.0622, therefore, on average the mean S-scores from the bootstrapped data differed by only 0.0622 when compared to the corresponding experimental S-score (Fig. 3A). The std for the S-scores within the non-curated dataset was 1.257. The MAE constitutes just 4.94% of the error, making the MAE a negligible difference. For the bootstrapped curated dataset, the MAE was 0.0657 and the std for the original curated dataset was 1.256 (Fig. 3B). The MAE equals just 5.23% of the std, again making the difference negligible, therefore demonstrating the robustness of the S-scores.

Both the non-curated and curated datasets produced robust and reliable S-scores. However, following dataset curation, it is imperative to benchmark the fitness scoring of the curated dataset against the non-curated dataset. Furthermore, it is important to confirm ChemGAPP Big is as effective as previous software. In order to do this, a cosine similarity analysis of phenotypic profiles was performed between genes from the same operon and those from different operons. ChemGAPP Big was equally proficient at fitness score assignment as E-MAP, which was employed within the Nichols et al. study looking at the *E.coli* KEIO collection. This held true for both the curated and non-curated datasets (Fig. 3C). The AUC for the curated and non-curated datasets were 0.6738 and 0.6736, respectively, versus an AUC of 0.6797 for Nichols et al. Since some genes in the same operon may not have the same function or may have directly opposite functions, this measure is not a perfect model. Therefore, to further prove the effectiveness of ChemGAPP Big, it was vital to explore if biologically relevant phenotypes were still displayed within the dataset.

### 3.3 ChemGAPP Big displays hits with biological significance

**Figure 4:**
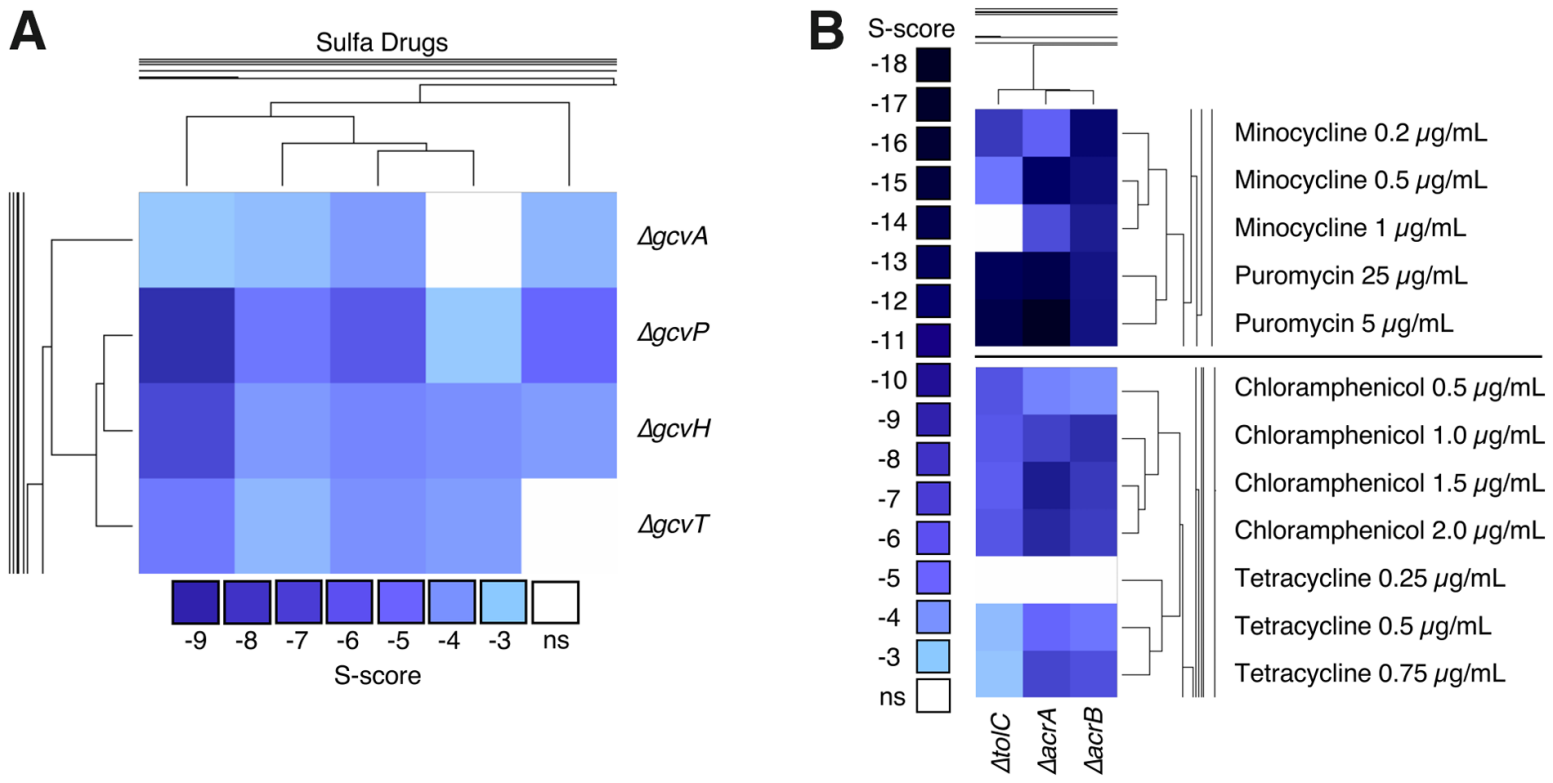
Clustered heatmaps displaying the S-scores for various single gene knockout mutants in different conditions. (A) The GCV system in sulfonamide drugs (Sulfa), from left to right: Sulfamonomethoxine 100 µg/ml; Sulfamethoxazole 100 µg/ml; Sulfamethoxazole 200 µg/ml; Sulfamethoxazole 300 µg/ml; Sulfamonomethoxine 50 µg/ml. (B) AcrAB-TolC system mutants in presence of AcrAB-TolC substrates. ns, non-significant.

The major aim of ChemGAPP Big is to produce datasets which are able to accurately reveal biologically relevant phenotypes. Therefore, it was crucial that when reanalysing the Nichols et al., dataset with ChemGAPP Big that the biologically significant hits were retained. Within the Nichols et al. (2011), paper, they discovered that mutants within the GCV system were susceptible to sulfonamides. The GCV system is one of two branches responsible for the conversion of tetrahydrofolate (THF) to 5,10-methylene THF, an essential process within the THF biosynthesis pathway. Nichols et al. (2011) performed validation experiments and determined this to be a true phenotype in the dataset. This phenotype was retained within the ChemGAPP Big dataset, with significant S-scores between −3 and −9 (Fig. 4A).

Another means of validating the accuracy of the S-scores is by searching for known synthetic lethal pairs. For example, efflux pumps are one of the major drug resistance mechanisms within bacteria. In *E. coli*, the major RND efflux pump system responsible for exporting many classes of antibiotics, including tetracyclines and chloramphenicol, is the AcrAB-TolC system (Elkins and Nikaido 2002; Chowdhury et al. 2019). Thus, we can assume that if this pump is deleted, the cell will be sensitive to its substrates. Following S-score analysis by ChemGAPP Big, we confirmed that Δ*acrA*, Δ*acrB*, and Δ*tolC* were in fact sensitive to the systems substrates, with significant negative S-scores between −3 and −18 for minocycline, puromycin, chloramphenicol, and tetracycline (Fig. 4B).

The significant S-scores for both the GCV system and the AcrAB-TolC system further provide evidence that ChemGAPP Big is capable of producing accurate S-scores for successful hit acquisition.

### 3.4 ChemGAPP Small produces informative fitness ratios

**Figure 5:**
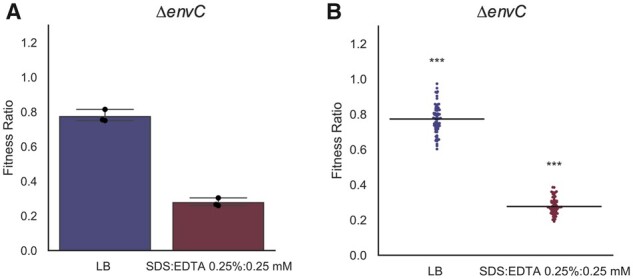
ChemGAPP Small produces informative fitness ratios. ChemGAPP Small provides the option of two different output plot types. (A) Bar plot output of ChemGAPP Small for Δ*envC* in LB, and 0.25% SDS + 0.25 mM EDTA, error bars represent 95% confidence intervals. (B) Swarm plot output of ChemGAPP small for Δ*envC* in LB, and 0.25% SDS + 0.25 mM EDTA. ns = *P* > 0.05; ***0.0001 < *P* ≤ 0.001.

To validate ChemGAPP Small a screen was performed based on the observation within the Nichols KEIO chemical genomics dataset that Δ*envC* showed decreased fitness in membrane perturbing stresses (Nichols et al. 2011). Within the Nichols dataset, Δ*envC* within 0.5% SDS + 0.5 mM EDTA had an S-score of −8.5, showing a significant decrease in fitness. In the current study, Δ*envC* was grown on LB and LB + 0.25% SDS + 0.25 mM EDTA. Within both conditions, a statistically significant decrease of Δ*envC* fitness was seen compared to the wildtype (Fig. 5). However, this decrease was more severe in the SDS + EDTA condition, with a fitness ratio of 0.28 compared to 0.77 on LB. This indicated a similar response to that observed in the Nichols et al. chemical genomic screen. Demonstration of this response by ChemGAPP Small, provides evidence for its effectiveness at interpreting small-scale screen data into informative fitness ratios.

### 3.5 ChemGAPP GI successfully reveals genetic interaction types

**Figure 6:**
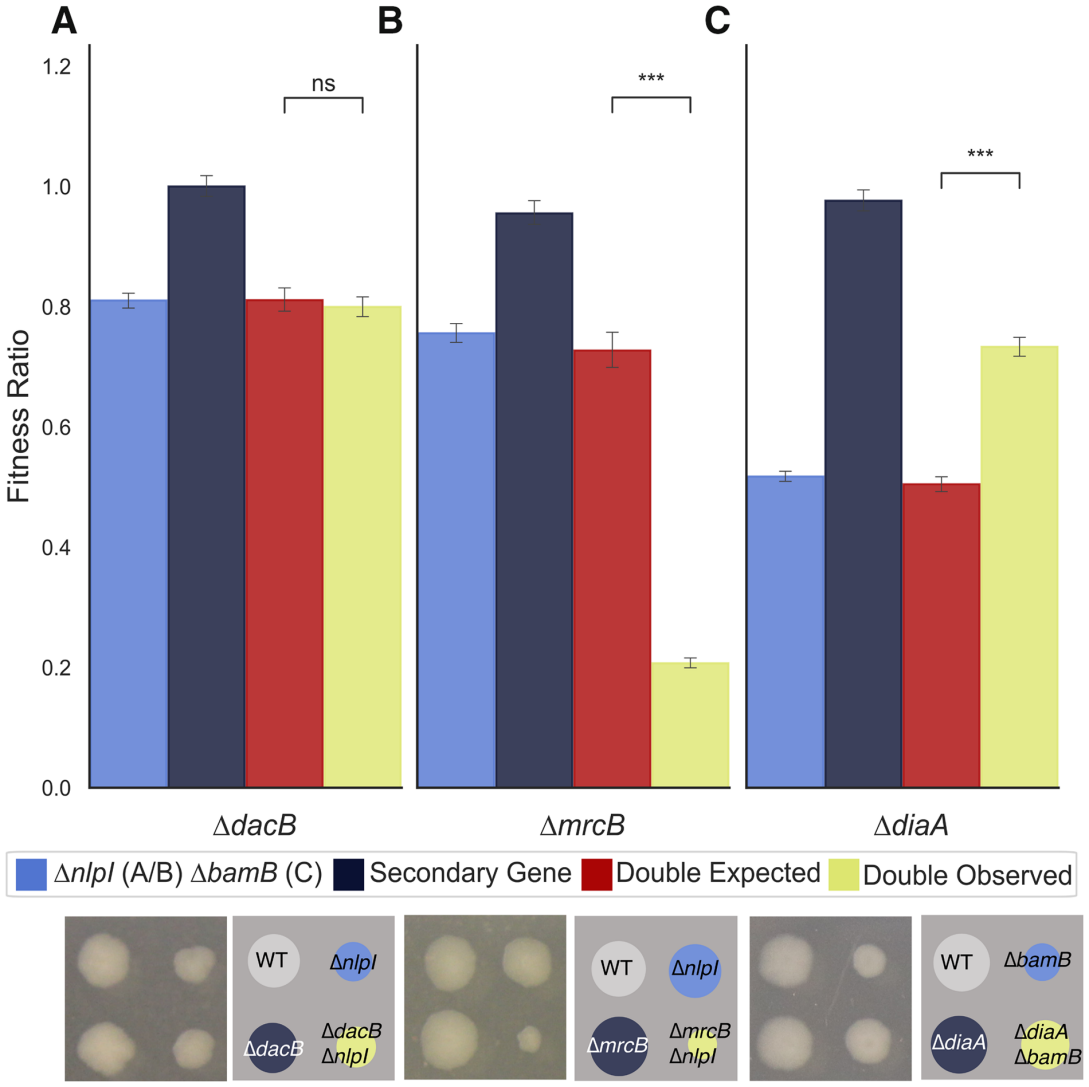
ChemGAPP GI can accurately predict all types of genetic interactions. (A) Nonsignificant difference between the double expected and double observed fitness ratios, therefore indicating no epistasis between *dacB* and *nlpI*. (B) Double observed significantly lower than double expected, showing negative epistasis between *mrcB* and *nlpI*. (C) Double observed significantly fitter than double expected. Showing positive epistasis between *diaA* and *bamB*. ns = *P* > 0.05; ***0.0001 < *P* ≤ 0.001.

To validate ChemGAPP GI, we reanalysed data from a previous study evaluating the genetic interactions between the outer membrane (OM) lipoprotein *nlpI* and peptidoglycan machineries (Banzhaf et al. 2020). Banzhaf et al. (2020) showed that *nlpI* did not genetically interact with the penicillin-binding protein 4 encoding gene *dacB*. Here, the difference between the double expected fitness ratio and the double observed ratio was 0.002. However, *nlpI* showed a synergistic interaction with PBP1B gene *mrcB*, with a difference of 0.62 between the expected and observed ratios (Banzhaf et al. 2020). ChemGAPP GI successfully reproduced these results using the raw data from the Banzhaf et al. study, showing differences in the double expected and double observed ratios of 0.011 and 0.52 for *dacB* and *mrcB*, respectively (Fig. 6A and B). Further to this, we validated a previously described positive genetic interaction between the OM lipoprotein *bamB* and DNA replication activator *diaA*. After analysis with ChemGAPP GI, evidence of a positive genetic interaction was achieved (Fig. 6C). ChemGAPP GI, is consequently capable of accurately predicting all types of genetic interactions.

## 4 Discussion

ChemGAPP was designed to address the need for a chemical genomics analysis software that is easy to use and fully suited to purpose. The current lack of software has led to the implementation of in-house scripts, often based on EMAP Toolbox (Shiver et al. 2016, 2021). This gap in the field limits the analysis pipeline to bioinformaticians and computational biologists, who are able to write and implement these scripts. One recent study, however, produced an analysis package (ScreenGarden) for plate-based high-throughput screens to fill the gap of deprecated analysis software (Klemm et al. 2022). However, similar to EMAP Toolbox, this software was not specifically designed for chemical genomic screen data and was tested on Synthetic Physical Interaction (SPI) screen data (Klemm et al. 2022). Furthermore, ScreenGarden produces log growth ratios and Z-scores which are compared to one or two control plates as its fitness measure. For the analysis of chemical genomic screen data, this is not as effective as using S-scores, which consider the effect of gene mutation across all conditions. Despite this, in instances where fitness ratios are more applicable, such as small-scale screens, ChemGAPP Small covers this requirement. This makes ChemGAPP a more comprehensive tool and, considering the nature and aim of chemical genomic screens, more suitable for chemical genomic analysis.

## 5 Conclusions

We have introduced ChemGAPP a comprehensive, user-friendly wrapper software dedicated to the analysis of a variety of chemical genomic screen types. ChemGAPP’s three sub-packages were specifically designed to allow a wider scientific audience to engage in chemical genomic techniques by streamlining each dedicated analysis pathway.

## Supplementary Material

btad171_Supplementary_Data

## Data Availability

The data underlying this article will be shared on reasonable request to the corresponding author.
