# Supplementary material for: ChemGAPP: a tool for chemical genomics analysis and phenotypic profiling
Source: Bioinformatics. 2023 Apr 4;39(4):btad171. doi: 10.1093/bioinformatics/btad171 (PMC10085634; doi:10.1093/bioinformatics/btad171)
Supplement: btad171_Supplementary_Data [file btad171_supplementary_data.zip › Additional_File_1.pdf]

Supplementary Figures

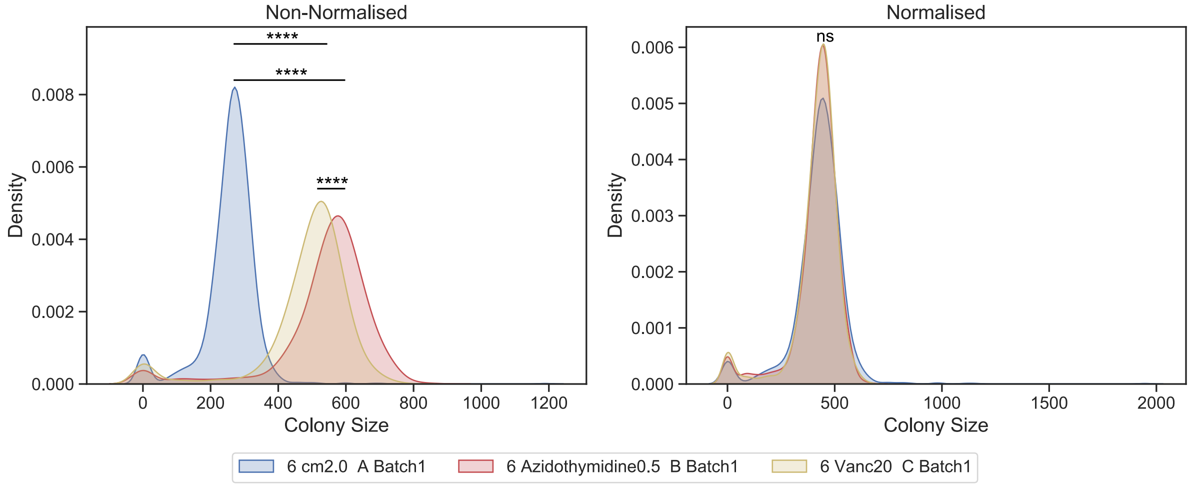

Figure S1: Density plot showing the difference between pre and post normalised colony sizes for three randomly selected condition plates. Normalisation scales colony sizes and reduces differences due to condition related growth effects. Upon normalisation, plates become more uniform, and significant differences between distributions are lost. Cm2.0 = Chloramphenicol 2 µg/mL; Azidothymidine0.5 = Azidothymidine 0.5 ng/mL; Vanc20 = Vancomycin 20 µg/mL. **** : p-value <= 1.00e-04, ns: p-value > 0.05.

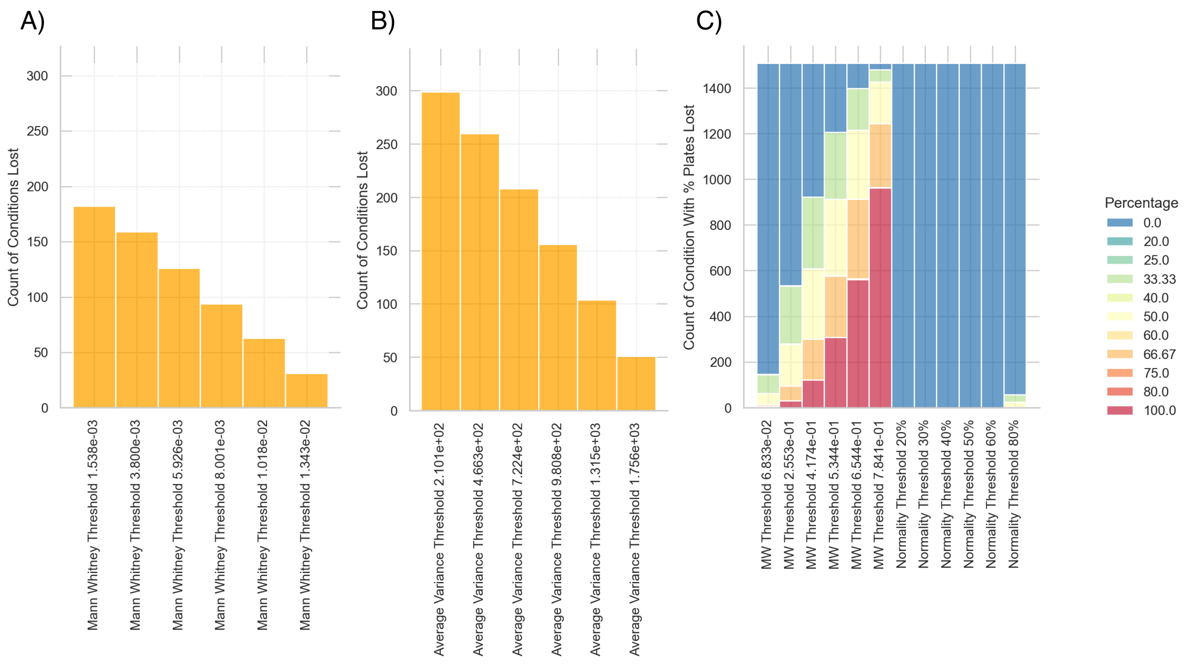

Figure S2: Threshold selection bar plots outputted by ChemGAPP Big. ChemGAPP makes threshold selection simple, with informative plots which display the quantity of data lost at various threshold for: A) Mann-Whitney Condition Level Analysis; B) Condition Level Variance Analysis; C) Mann-Whitney Plate Level Analysis and Z-score analysis.

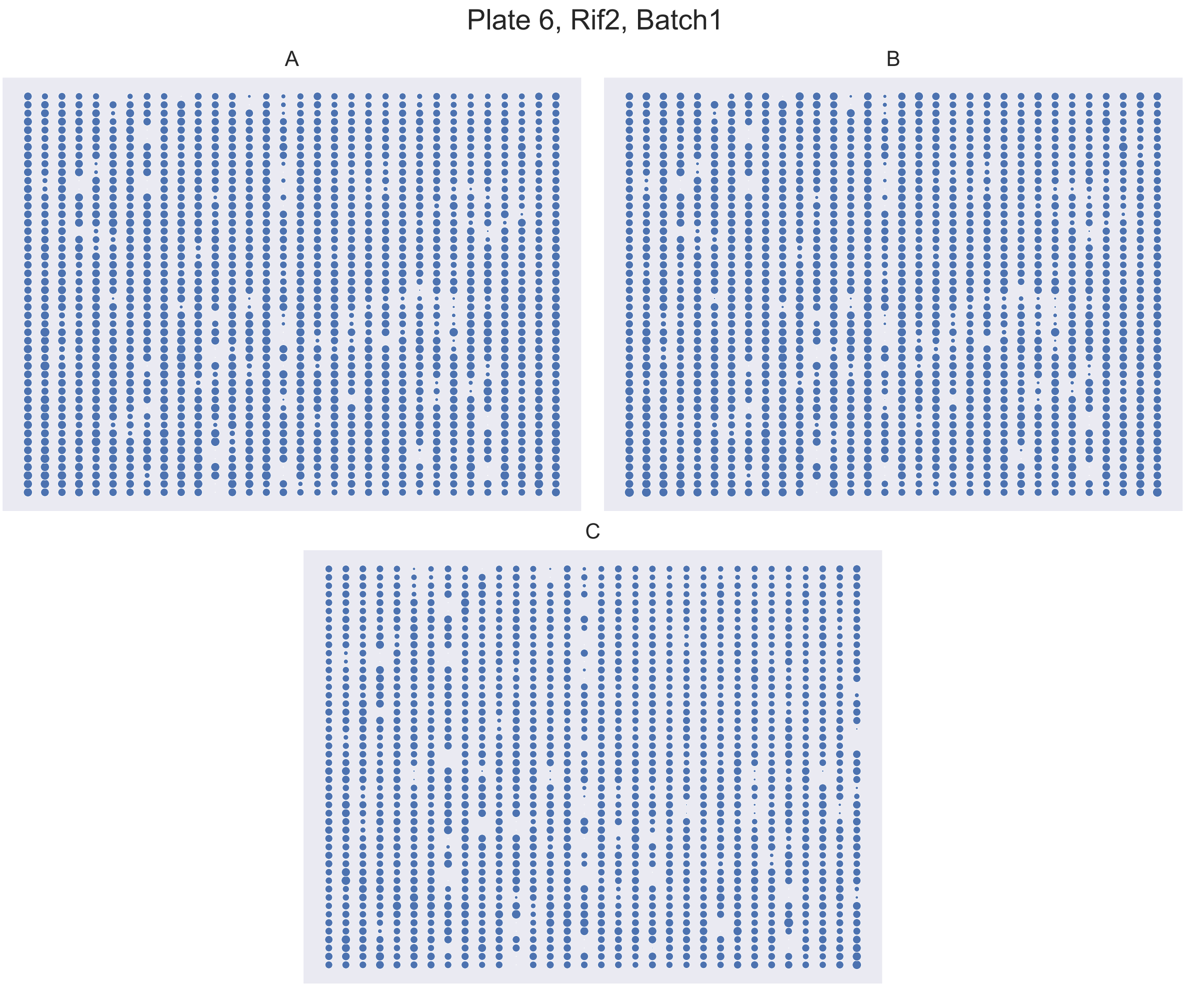

Figure S3: Replicate C highlights defects of missed and unequal pinning. Plate matrix depicting the colony sizes within replicate plates of the condition Rif2 (Rifampicin 2 µg/mL), Plate 6, Batch1. Replicate C shows an increased number of missing colonies (21) compared to A (4) and B (4). The upper segment of replicate C has generally smaller colonies than the lower segment, highlighting unequal pinning.
